# Klar entscheiden, transparent führen und eindeutig begeistern

**DOI:** 10.1365/s35764-021-00321-2

**Published:** 2021-03-15

**Authors:** Johanna Dahm

**Affiliations:** 1 Lindenallee 13, 53173 Bonn, Deutschland; 2 Universität zu Köln, Köln, Deutschland

Führung und Wertschätzung tragen nicht nur in Krisenzeiten maßgeblich zum Employer Branding von Unternehmen bei, sondern zählen zu den wirtschaftlichen Erfolgsfaktoren. Doch in den meisten Fällen bleiben sie nicht mehr als ein Lippenbekenntnis.

Dies bestätigt eine Umfrage zum Thema *Führungsleitbild & Kultur* mit ca. 200 Unternehmern, Führungskräften sowie leitenden Angestellten. Dr. Johanna Dahm, internationale Beraterin & Organisationsentwicklerin, führte die Befragung und kommentierte: „Die meisten Verantwortlichen sind führungsmüde und erwarten gerade von neuen, aber auch Bestandsmitarbeitern begeisternde Ideen, die zu Neukundengewinnung, Kapitalbeschaffung und einem konstanten Cashflow beitragen. Oft sind die Leader selbst aber gar nicht zur Motivation bereit. Tatsächlich selbst als belastbares Vorbild und Coach zu fungieren, haben die meisten Vorgesetzten nie gelernt. 76 % der Mitarbeiter wünschen sich in ihrem Vorgesetzten einen Coach oder Mentor, und das nicht nur in Zeiten des Wandels. Wer die Bedarfe von Mitarbeitern und Teams bereits erkannt hat oder spätestens jetzt erkennt und wahrnimmt, der sichert Motivation und Begeisterung.“

Die Ausbildung eines deutschen Managers fällt im internationalen Vergleich mit durchschnittlich 7 Tagen pro Jahr um die Hälfte kürzer aus als in anderen Ländern. Zwar zahlen deutsche Unternehmen mit ca. 2278 € pro Kopf doppelt so viel wie Arbeitgeber anderer Länder, die wiederum nur ca. 1200 € investieren. Das geringere zeitliche Investment in Deutschlands Führungskultur korreliert mit einem niedrigen Führungsindex: Führungskräfte werden durch ihre Mitarbeiter schlecht bewertet, wenn es um emotionales, soziales und auch ethisches Verhalten geht. Gerade in Zeiten des Umbruchs, wie unter Covid-19, stehen die Charaktereigenschaften von CEOs, Inhabern von Schlüsselpositionen und allen Managern auf dem Prüfstand.

Was konkret zeichnet nun führungsfähige Vorgesetzte aus, wie kommunizieren, kollaborieren und kooperieren diese? Und wie können Unternehmen JETZT Führung und Begeisterung seitens des Managements steigern, um Erfolgsfaktoren wie Sicherheit, Zufriedenheit, aber auch Leistungsbereitschaft sicherzustellen?

Viele Manager seien krisenträge geworden, darum müsse Führungsbereitschaft und Mitarbeiterorientierung zu einem Managemententschluss werden. „Wir müssen wieder Begeisterungsfähigkeit lernen. Gerade weil aktuell alle Betriebe den Anforderungen bestehender und neuer Märkte mit vereinten Kräften begegnen müssen, zeigt sich, wer Mitarbeiter motivieren, binden, halten kann und wer eben nicht. Nur weil andere Unternehmen bereits in Leadership investiert haben, ist das kein Argument, jetzt nicht damit anzufangen. Und die Begeisterung der Mitarbeiter ist nun einmal der Index, ob Markt- und Kundenbedürfnisse getroffen werden und das Unternehmen überlebt“.

Die meisten Führungskräfte seien keine Entscheider und setzten die verkehrten Prioritäten, „9 von 10 treffen gar keine Entscheidungen und agieren wie Fachkräfte“. Umgang mit Personal, Delegation und Vertrauen seien kaum ausgebildet, die strategische Weitsicht fehle. Daraus resultieren die konstant schlechten Mitarbeiterbewertungen, der fragwürdige Ruf bei Kunden, die hohe Fluktuation und die grundsätzliche Demotivation. Führungs- und Businessmodelle müssen komplett neu gedacht werden, um Menschen zu motivieren und Chancen im Change Management zu nutzen.

## Erst entscheiden, dann agieren

Insgesamt 86 % der Mitarbeitenden fühlen sich durch ihre Vorgesetzten überfordert. Sie beklagen den Umgangston, aber vor allem die internen Systeme und die Delegation sowie die Nichteinhaltung klarer Kommunikation. Trotz digitaler Möglichkeiten stecken viele Unternehmen hier noch in den Kinderschuhen, nutzen Services weder zur transparenten internen Arbeitserleichterung noch zur Abstimmung mit Kunden. „Immer häufiger brechen Kandidaten ihrerseits Bewerbungsverfahren ab, weil sie die Infrastruktur des Unternehmens als rückständig erkennen und gehen zum Wettbewerb. Hier gehe es nicht um das Gehalt, sondern um Entfaltungsmöglichkeiten.

Der Arbeitgeber solle sich dringend die Frage stellen, wie er gleich beim Bewerbungsgespräch eine Candidate Experience schafft, den Kandidaten begeistert, im Unternehmen ein dauerhaftes Umfeld generiert, das konkurrenzfähig bleibt. Das sei auch wirtschaftliche Verantwortung und Zukunftssicherung für Mensch und Unternehmen. Aber die meisten Entscheider machen da noch immer einen Rückzieher. Natürlich sollen Unternehmen ihren Wettbewerbsanspruch nicht verlieren, aber die persönlichen Merkmale des Managements bedürfen der Generalüberholung: intellektuell, emotional, sozial und eben auch ethisch“, mahnt Dahm.

Die Diskrepanz zwischen Sagen und Machen, zwischen dem Erkennen von Missständen und Umsetzen von Zusagen mache Unternehmen gegenüber Kunden und Kandidaten jedoch immer unberechenbarer. Man müsse endlich damit aufhören, uns Bilanzen und Nichthandeln schön zu reden und damit Führungsschwäche zu entschuldigen. Es gehe darum, dass stattdessen Ehrlichkeit und Entschlusskraft zu Unternehmenswerten erhoben werden. Dass die Top-3-Agenda-Punkte – Demografie, Digitalisierung und Diversität – in Deutschlands Unternehmen so vernachlässigt werden, sei eine Unterlassung und komme auf viele Betriebe jetzt wie ein Bumerang zurück: „Seit Ende der 90er-Jahre sprechen wir über diese Themen und warten auf ein Wunder, einen Beschluss von oben“, sagt Dahm, „dabei sind Betriebe für die Beschäftigungsbefähigung und den Wissenstransfer aller Generationen, die dauerhafte Innovation und die Einbindung von Kundenbedürfnissen weit über die Geschlechterfrage hinaus eindeutig selbst verantwortlich.“

## Return on Investment neu definieren

Profitabilität müsse durch die Entwicklung einer Führungskultur, die Talententwicklung und Delegation von Verantwortlichkeiten konsequent auf- und ausgebaut werden. Wer die Chance in Führung und Begeisterung erkenne, der müsse das aktiv und nachhaltig tun. Maßnahmen dürfen keine Lippenbekenntnisse oder halbherzig verfolgte Initiativen bleiben, sondern als Entscheidung kommuniziert, in Teams mit Ideen und Entschlusskraft manifestiert und auch dem Kunden gegenüber spürbar gemacht werden.

Dabei dürfe nicht zurückgerudert werden, selbst wenn es in den Anfängen schwerfallen dürfte. Natürlich behaupten die meisten Führungskräfte, mitarbeiterorientiert zu sein, meinen damit aber die nach dem Gießkannenprinzip verteilten Trainings, den Tankgutschein, das on top vergebene Projekt und natürlich die Weihnachtsfeier. Diese Auflistung ist typisch für über 90 % der deutschen Unternehmen und skizziert das Problem: Zu wenigen Mitarbeitern biete sich die Chance auf eine Entwicklungs- oder gar Laufbahnplanung, allenfalls werde sie durch zusätzliche Programme ausgebrannt. Erfolge gehen im Betriebsalltag unter, der Fokus liegt auf Tagesumsatz und Fehlerbehebung. „Führungskultur muss dringend zum messbaren Erfolgsfaktor einer jeden Firma werden, an dem sich der ROI ausrichtet“. Die Aktualität: Krankheitstage steigen wegen psychischer Überforderung, vernachlässigter Delegation, mangelnder Führungsqualifikation, was Kosten in Milliardenhöhe produziert.

Neben der Führungs- und Talententwicklung gibt es weitere Felder, in denen Führungskräfte umdenken müssen: Der Fokus sollte sich von Ansagen dringend aufs Zuhören und auf die unternehmerische Verantwortung an sich verlagern: „Was wollen und können meine Mitarbeiter, welchen Beitrag zum Erziehungs‑, Gesundheitssystem und zur Umwelt kann ich als Unternehmer beitragen – das sind die Fragen, die auf der Agenda stehen sollten“. Eine solche Fokusverschiebung in der CEO- und Führungs-Agenda verlangt Einfühlungsvermögen und Empathie, sowohl den Mitarbeitern als auch Kunden und Marktbegleitern gegenüber.

Gerade in männerdominierten Betrieben werden diese Kompetenzen noch als sehr schwach und als vernachlässigbar angesehen. Unternehmen mit Frauen im Management schneiden besser ab, sind von Insolvenzen und Disruptionsrisiko weniger betroffen. Das Umdenken gehe aber zu langsam, dass erst 21 % der Unternehmer, deren Betriebe zur Nachfolge anstehen, überhaupt eine Frau als Nachfolgerin in Erwägung ziehen, trotzdem hervorragend ausgebildete weibliche Kräfte intern und extern zur Verfügung stehen. „Wir brauchen ein gemischtes Topmanagement, um die Bedürfnisse von Menschen zu begreifen. Das müsse jetzt Interesse eines lernwilligen Vorstands sein“.

## Zu langsam für den Wettbewerb

Beim Thema Lernwilligkeit gäbe es jedoch eine deutliche Diskrepanz zwischen „talking and doing“, zwischen dem, was gesagt und wirklich gemacht wird: „Unternehmen sind kaum und von Herzen wirklich entschlossen, ihren Mitarbeitern wirklich zu vertrauen, klar zu delegieren, Erfolge zu feiern, geschweige denn Frauen zu rekrutieren. Das sehen wir allein daran, dass 30 % weibliche Kandidaten allein in den MINT-Berufen unberücksichtigt bleiben“. Dahm konstatiert, dass es an letzter Konsequenz, an Ehrlichkeit und Entschlusskraft mangele, weil die Angst davor zu groß ist, Gewohnheiten aufzugeben, vergangene Erfolge nicht wieder aufleben lassen zu können. „Da scheint das Neue einfach zu neu“.

Betrachte man den internationalen Kontext, sehe man, dass das den Innovationsstandort Deutschland und auch die Freiheit des Unternehmertums immer mehr schwäche. Während Skandinavien oder auch die Schweiz mit massiven Investitionen das Innovationstempo fördern, schrumpft in Deutschland die Bereitschaft zur Flexibilität, wobei sich Unternehmen zumindest zum Teil hinter Argumenten der Bürokratie und Komplexität verstecken. Natürlich ist es so, dass Auflagen etwa für Gründungen oder Patentanmeldungen in Deutschland zu hoch sind und oft die Möglichkeiten gerade kleiner Unternehmen übersteigen. Zudem dauern Anträge und Verfahren zu lange und verzögern die wirtschaftliche Verwertbarkeit von Innovationen.

